# AN ANALYSIS OF THE POTENTIAL ROLE OF CHEST TOMOSYNTHESIS IN OPTIMISING IMAGING RESOURCES IN THORACIC RADIOLOGY

**DOI:** 10.1093/rpd/ncw040

**Published:** 2016-06-07

**Authors:** Cecilia Petersson, Magnus Båth, Jenny Vikgren, Åse Allansdotter Johnsson

**Affiliations:** 1 Department of Radiology, Sahlgrenska University Hospital, SE-413 45 Gothenburg, Sweden; 2 Department of Radiation Physics, Institute of Clinical Sciences, The Sahlgrenska Academy at University of Gothenburg, SE-413 45 Gothenburg, Sweden; 3 Department of Medical Physics and Biomedical Engineering, Sahlgrenska University Hospital, SE-413 45 Gothenburg, Sweden; 4 Department of Radiology, Institute of Clinical Sciences, The Sahlgrenska Academy at University of Gothenburg, SE-413 45 Gothenburg, Sweden

## Abstract

The aim of the study was to investigate the potential role of chest tomosynthesis (CTS) at a tertiary referral centre by exploring to what extent CTS could substitute chest radiography (CXR) and computed tomography (CT). The study comprised 1433 CXR, 523 CT and 216 CTS examinations performed 5 years after the introduction of CTS. For each examination, it was decided if CTS would have been appropriate instead of CXR (CXR cases), if CTS could have replaced the performed CT (CT cases) or if CT would have been performed had CTS not been available (CTS cases). It was judged that (a) CTS had been appropriate in 15 % of the CXR examinations, (b) CTS could have replaced additionally 7 % of the CT examinations and (c) CT would have been carried out in 63 % of the performed CTS examinations, had CTS not been available. In conclusion, the potential role for CTS to substitute other modalities during office hours at a tertiary referral centre may be in the order of 20 and 25 % of performed CXR and chest CT, respectively.

## INTRODUCTION

Chest tomosynthesis (CTS) is a relatively new technique of acquiring several low-dose projection radiographs over a limited angular range and using these projection radiographs to reconstruct contiguous section images of the chest. The modality offers an increased sensitivity regarding detection of pulmonary pathology in comparison with chest radiography (CXR) at a modest increase (<0.1 mSv) in radiation dose^(1–3)^. Independent studies on the topic of nodule detection have shown an overall 3-fold increase in sensitivity for CTS in comparison with CXR^(4–7)^, indicating the possibility of using CTS in clinical practice for detection of pulmonary metastases^(6, 7)^, as well as for improving the performance in revealing pulmonary mycobacterial disease^(8)^. Furthermore, there is scientific evidence that CTS is decisive for confirming or ruling out pathology suspected on CXR^(9, 10)^ and that clinical implementation of CTS results in reduced costs for diagnostic imaging^(11)^. Additionally, CTS has been suggested as an imaging alternative to computed tomography (CT) in the follow-up of patients with cystic fibrosis, taking the superior sensitivity compared with CXR in detecting pathology and the lower radiation dose compared with CT into account^(12, 13)^. Concerning other pulmonary pathology, CTS has been reported as superior in comparison with CXR regarding detection of emphysema^(14)^ and asbestos-related changes^(15)^. On the other hand, there are indications that the sensitivity of CTS decreases with diminishing size of the pathology and with drop off in CT attenuation value^(16)^. Consequently, ground-glass opacities have been reported as a potential pitfall for the new modality^(17, 18)^.

There are reports in the scientific literature stating that CTS may avoid the need for CT in 70–80 % of patients presenting with suspected lesions on CXR^(9, 19)^. However, to the knowledge of the authors, no study has investigated the impact of CTS on the workflow at a department of thoracic radiology. The present study was therefore conducted to investigate the actual role of CTS as well as the potential role of the modality, i.e. to what extent the modality may substitute CXR and CT. The investigation was based on a survey of examinations performed 5 years after the introduction of CTS in clinical praxis at a tertiary referral centre.

## MATERIALS AND METHODS

The study was approved by the regional ethical review board, and the survey was conducted by a radiology resident (C.P.) with almost 5 y of experience in radiology, supervised by a senior consultant in thoracic radiology (Å.A.J.) with 23 y of experience in radiology, including 18 y of experience in thoracic radiology and substantial experience in CTS.

### The studied department and examinations

The studied department of thoracic radiology is part of a tertiary referral centre with national healthcare assignment regarding heart and lung transplantations as well as thoracic surgery for adults with congenital heart disease. The hospital is also the regional trauma centre.

CTS was introduced in clinical praxis in December 2006. The survey was conducted in January 2015, and in order to achieve a follow-up time of at least 2 y regarding the performed CTS, a search for examinations performed during 2012 was made in the radiology information system (RIS). Thus, the survey reflected the use of CTS 5 years after the introduction of CTS in clinical praxis. In order to avoid the influence of holidays, the third week in 5 consecutive months (August–December 2012) was chosen and all CXR [including a posteroanterior (PA) and a lateral projection], chest CT and CTS examinations carried out during office hours (7 a.m. to 5 p.m.) were registered.

### Data collection

For all modalities, patient age, sex and indication for the examination were recorded. It was also noted whether another CT examination was combined with the chest CT [i.e. brain, neck, abdomen or positron emission tomography (PET)]. Concerning the performed CTS examinations, more detailed information was retrieved: (1) whether it was a clinical or radiological referral (the former refers to an examination that has been requested by a referring clinician, whereas the latter refers to an examination that has been requested by a radiologist after evaluating a CXR); (2) if the pathology of interest for the CTS examination was known from any previously performed CT; (3) if CT had been performed during the follow-up period; (4) the follow-up time after the performed CTS, which was determined from information available in the RIS; (5) the presence of disturbing artefacts in the CTS examination, commented on in the radiological report; and (6) whether CTS was considered adequate given the information prior to the examination. Furthermore, the survey included a search for potentially adverse events, i.e. pathology not described in the CTS report that was detected by a CT performed during the follow-up period and that could matter to patient treatment.

### Determining the role of chest tomosynthesis

By means of predefined criteria (see below), based on scientific evidence in addition to experience from clinical praxis, and information available in the RIS (referrals and reports) as well as in the picture archiving and communication system (PACS) (images), prior to the performed examination, it was for each case determined (1) if CTS would have been appropriate instead of the performed CXR, (2) if CTS could have replaced the performed CT and (3) if CT would have been performed had CTS not been clinically available.

#### Chest tomosynthesis substituting chest radiography

The following indications were used as criteria for deciding that a CTS would have been appropriate instead of the performed CXR: pulmonary metastases; follow-up of pathology known from CT, judged not visible in CXR; pathology suspected on previous CXR; cystic fibrosis; bronchiectasis; emphysema; asbestos-related changes; interstitial lung disease (ILD); pulmonary sarcoidosis; and cases where the CXR was anticipated to be difficult to interpret because of pleural plaques. The indication tuberculosis was not used as a criterion due to the low incidence in the region where the study was conducted^(20)^.

#### Chest tomosynthesis substituting computed tomography

The main criterion used for determining whether a CTS could have replaced a conducted CT was that the referral concerned only pulmonary pathology deemed to be visible on CTS, comprising indications such as follow-up of pulmonary opacities including nodules, cystic fibrosis, bronchiectasis, pulmonary sarcoidosis, ILD and pneumothoraxes. CT examinations where the detection of ground-glass opacities was of importance were not considered replaceable by CTS.

#### Computed tomography substituting chest tomosynthesis

Regarding whether the performed CTS would have been substituted by a CT or not, had CTS not been available in clinical praxis, the following criteria were used. Clinical referrals where the pathology of interest could not be visualised by CXR and radiological referrals with suspicion of pathology on CXR, where the radiologist could have not given a definite report without the CTS, were both categorised as having been replaced by CT. A typical example of such a radiological referral was a suspected pulmonary lesion on CXR, which by CTS was ruled out as osseous, a scenario that without clinically available CTS would have been further investigated by CT.

## RESULTS

### Description of the examinations

A total of 2172 examinations had been conducted during office hours of the 5 weeks selected: 1433 CXR (714 women, 12–96 y), 523 CT (288 women, 16–95 y) and 216 CTS (125 women, 18–92 y). There were 60 different indications for the examinations performed. CT of the chest was performed in conjunction with another CT or PET in 53 % of the cases.

Regarding CTS, there was a clinical referral for 53 % of the examinations. The pathology of interest for the CTS examinations was known from previously performed CT in 48 % of the cases and in 39 % a CT was performed during the follow-up period. The follow-up time for the patients ranged from 0 to 29 months, and 88 % of the patients undergoing CTS had a follow-up of at least 24 months (12 % of the patients died during the follow-up time). Artefacts were found in 6 % of the CTS examinations, the main cause being insufficient breath holding. One patient had difficulties standing still, and one patient had undergone embolisation of an arteriovenous malformation, where the embolisation material caused artefacts. The CTS examinations were considered appropriate in 96 % of the cases (208 of 216). Examinations considered as inappropriate included ground-glass opacities, investigation of a pericardial cyst and patients exhibiting language difficulties (not understanding breath-holding instructions).

One potentially adverse event was found: a case where CTS failed to depict ground-glass opacities proved by CT 3 months prior to the CTS. A follow-up CT performed 2 months after the CTS depicted the ground-glass opacities with exactly the same distribution as on the previous CT.

### Analysis of the performed examinations

#### Chest tomosynthesis replacing chest radiography

Concerning the CXR examinations, it was judged that CTS would have been appropriate in 219 cases (15 % of the cases), where the top three indications were found to be metastases (35 %), sarcoidosis (25 %) and ILD (16 %).

#### Chest tomosynthesis replacing computed tomography

CTS could be considered as an alternative to the conducted CT in 38 cases (7 %), where the main indication was ILD. Other indications included were follow-up of pulmonary nodules, characterisation of pulmonary opacities detected on CXR and bronchiectasis. Of the 38 performed CT examinations where CTS had been a possible substitute, 7 cases had another CT examination performed together with the chest CT. A part of CT as well as PET/CT referrals concerned work-up of pulmonary opacities, where a CTS indicating suspicion of a pulmonary malignancy already had been performed, and these examinations were not considered to be exchangeable with the new modality.

#### Probable computed tomography without chest tomosynthesis available

It was estimated that, had CTS not been clinically available, 136 cases (63 %) of the performed CTS would have been substituted by CT. The major indications were a suspected pulmonary lesion (25 %), pulmonary metastases (18 %) and nodule follow-up (13 %).

### The potential role for chest tomosynthesis to substitute chest radiography

An estimate of the accurate role for CTS in substituting CXR, based on the assumption that 291 CTS should have been performed [the appropriate CTS performed that would not have been substituted by CT (208–136 = 72) + the CXR cases where CTS was judged as being appropriate (219)] out of the 1505 (1433 + 72) CXR that would have been performed, had CTS not been available, generates that 19 % of performed CXR should be substituted by CTS.

### The potential role for chest tomosynthesis to replace computed tomography

**Figure 1.**
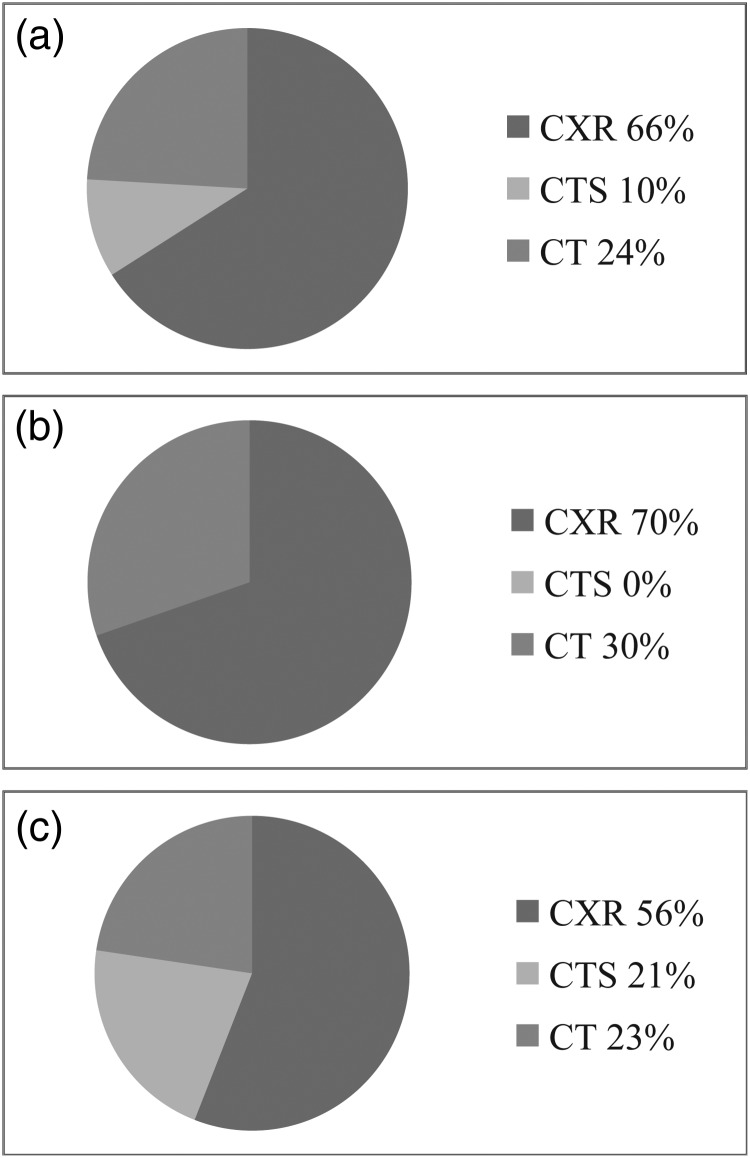
Pie charts showing the proportion of examinations (**a**) in the regular workflow of the studied radiology department, (**b**) in the event that CTS had been unavailable and (**c**) with the potential role of CTS replacing part of CXR and CT examinations. CXR, chest radiography; CTS, chest tomosynthesis; CT, computed tomography.

A calculation of the potential role for CTS to substitute CT based on the results of the present study yields that 659 chest CT examinations would have been performed (523 + 136), had CTS not been clinically available. Additionally, 38 performed chest CTs were judged as being exchangeable with CTS, rendering a potential for CTS to replace 26 % (174/659) of the chest CTs. An illustration of the proportions of the examinations at different scenarios is given in Figure 1.

## DISCUSSION

In the present study, the potential role of CTS in optimising imaging resources at a thoracic radiology department at a tertiary referral centre was evaluated. The results indicate that after 5 years in clinical praxis, there was still unused potential for CTS, even though an increased use of CTS compared with what has previously been reported after 2 years in clinical praxis is implied^(21)^. However, it must be remembered that the percentages found in the present survey reflect the environment and workflow of the studied department. CTS accounted for ∼10 % of the total number of examinations, whereas CXR and CT represented 66 and 24 % of the total number of examinations, respectively. One potentially adverse event among the 216 CTS was registered, a frequency in line with a previous study reporting two adverse events in 149 CTS examinations^(19)^. The case reported in the present study concerned ground-glass opacities, a pathology that has been described as a potential pitfall for CTS^(16–18)^. Nearly all performed CTS were considered appropriate (96 %), indicating an awareness of the limitations of the new modality at the studied department.

Tuberculosis is an indication that has been shown to benefit from the increase in sensitivity provided by CTS^(8)^. However, the incidence is low in the region^(20)^ of the tertiary referral centre studied. Replacing all CXR, performed under the indication of ruling out tuberculosis, with CTS would raise the proportion of CXR where substitution by CTS would be considered appropriate from 15 to 20 %. However, before doing so, the gain in diagnostic accuracy regarding the presence of pulmonary tuberculosis in a population at low risk of having this disease must be judged against the modest increase in radiation dose from the CTS examinations.

There is evidence that replacing CXR with CTS in search for metastases will render a significant increase in detection rate^(4–7)^, and search for pulmonary metastases was found to be the major indication where CTS was suggested as a substitute for CXR. In analogy with the reported improved sensitivity in the recognition of emphysema^(14)^ and clinical experience, examinations for pulmonary pathology due to sarcoidosis or ILD were also considered to benefit from the increase in conspicuity offered by CTS.

As part of a tertiary referral centre incorporating a department of thoracic surgery including heart and lung transplantations, a number of patients were examined repeatedly by CXR postoperatively, many not considered in need for the increased sensitivity offered by CTS. These repeated examinations reduce the proportion of CXR where replacement by CTS would be considered appropriate in comparison with other radiological departments where fewer patients are investigated repeatedly during the same week. Despite this, an increased use of CTS in the order of 20 % of performed CXR was judged as appropriate, indicating that the benefits of the new modality were not always under consideration.

Regarding the ability for CTS to replace a performed CT, a low proportion (7 %) was noted. This probably reflects that a significant proportion of the possible substitution by CTS already was occurring in clinical praxis. Even though only examinations performed during office hours were included in the study, a substantial part of the CT examinations was performed under indications not suitable for CTS, such as major trauma, pulmonary embolism and acute aortic syndrome. Including examinations performed 24/7 would lower the proportion where CTS could be regarded as an alternative to CT even further, the main indications for CTS in emergencies being difficult pneumothoraxes and detection of rib fractures in minor trauma^(2)^. Indications where CTS has been described or suggested as an alternative to CT in the literature comprise characterisation of opacities suspected on CXR^(9)^, follow-up of pulmonary nodules^(1–3)^ and surveillance of patients with cystic fibrosis (bronchiectasis)^(12)^. These indications were included as possible examinations to be exchanged by CTS, as well as selected cases performed under the indication ILD. CT examinations, where the detection of ground-glass opacities was of importance, were not considered replaceable by CTS^(16–18)^. It can be argued that detection of ILD has not been sufficiently studied. Excluding all ILD cases examined by CT and deemed as replaceable by CTS would reduce the proportion of CT, where CTS could be regarded as an alternative examination, to 6 %.

According to the clinical routine used, the patient stays at the radiology department until the CXR has been reviewed so that a supplemental CTS can be performed directly as a problem solving tool (radiological referral). The use of CTS for confirming or ruling out pathology suspected on CXR has previously been reported to reduce the need for CT by 70–80 %^(9, 19)^. A substantial part (63 %) of the performed CTS in the present study was being judged as a plausible substitute for CT, thereby probably saving radiation dose to the patients^(19)^ and imaging costs^(11)^, although these parameters have not been investigated in the current study. The proportion of performed CTS judged as probable CT without CTS available was of the same magnitude as in a previously conducted survey^(19)^.

The estimation of the potential role for CTS to replace CT indicated that ∼25 % of the CT examinations performed during office hours at the tertiary centre studied could be substituted by CTS. After 5 years in clinical praxis, CTS had replaced around 20 % of these examinations. The strength of the present study is that the CTS procedure is positioned in the context of other examinations in thoracic radiology, an approach that to the knowledge of the authors has not been reported previously in the scientific literature. The results may be of help for radiological departments that are considering installation of a CTS modality, to estimate what role CTS might play.

The major limitation of the present study is that it is a single-centre study. A multicentre survey including both tertiary and secondary centres would be preferable in order to directly generalise the results to radiology departments in different contexts. However, the predefined criteria presented may aid the translation of the results to the plausible impact at radiological departments in other contexts. Additionally, the use of predefined criteria, based on scientific evidence and long-term clinical experience of the new modality at the department, reduced the influence of the subjectivity of the observer performing the survey.

## CONCLUSIONS

The results of the present study indicate that 5 years after the introduction of CTS in clinical praxis, there was still room for CTS to substitute CXR and CT. The potential role of CTS to substitute other modalities during office hours at a tertiary referral centre may be in the order of 20 % of performed CXR examinations and 25 % of performed CT examinations.

## FUNDING

This work was supported by the Swedish Research Council (2011-488, 2013-3477), the Swedish Radiation Safety Authority (2012-2021, 2013-2982 ), the Swedish Federal Government under the LUA/ALF agreement (ALFGBG-136281, ALFGBG-428961) and the Health and Medical Care Committee of the Region Västra Götaland (VGFOUREG-81341, VGFOUREG-483951).
